# The bacterial etiology and antimicrobial susceptibility of lower respiratory tract infections in Vietnam

**DOI:** 10.1186/s12941-025-00818-3

**Published:** 2025-08-31

**Authors:** Tran Thi Ngoc Dung, Chau Vinh, Pham Hong Anh, Vo Kim Phuong Linh, Ha Thanh Tuyen, Pham Thanh Tam, Nguyen Phu Huong Lan, Truong Thien Phu, Nguyen Su Minh Tuyet, Pham Hong Nhung, Van Dinh Trang, Nguyen Thi Van, Quynh Nguyen, Nguyen Thi Thanh, Thomas Kesteman, H. Rogier van Doorn, Guy Thwaites, Pham Thanh Duy

**Affiliations:** 1 Molecular Epidemiology Group, Oxford University Clinical Research Unit, Ho Chi Minh City, Vietnam; 2 Hospital for Tropical Diseases, Ho Chi Minh City, Vietnam; 3 Cho Ray General Hospital, Ho Chi Minh City, Vietnam; 4 Nhan Dan Gia Dinh General Hospital, Ho Chi Minh City, Vietnam; 5 Ha Noi Medical University, Ha Noi, Vietnam; 6 Bach Mai Hospital, Ha Noi, Vietnam; 7 National Hospital for Tropical Diseases, Ha Noi, Vietnam; 8 Viet Duc Hospital, Ha Noi, Vietnam; 9 Centre for Tropical Medicine and Global Health, University of Oxford, Oxford, UK

**Keywords:** Lower respiratory tract infection, Nosocomial pneumonia, Ceftazidime-avibactam, Antimicrobial resistance

## Abstract

**Background:**

Lower respiratory tract infection (LRTI) remains the leading infectious cause of morbidity and mortality globally. Key bacterial pathogens include *Acinetobacter baumannii*,* Pseudomonas aeruginosa*,* Klebsiella pneumoniae*,* Escherichia coli*, *Staphylococcus aureus* and *Streptococcus pneumoniae*. This study examined the prevalence and antimicrobial resistance patterns of major bacterial pathogens from community- and hospital-acquired LRTIs across six major hospitals in Vietnam.

**Methods:**

Between January 2022 and May 2023, 1000 bacterial isolates were collected through an isolate-based surveillance. Species identification and antimicrobial susceptibility testing were performed by VITEK-2/Phoenix M50, with MICs determined by E-test or broth microdilution. Multiplex PCRs were used to detect common AMR genes.

**Results:**

*A. baumannii* (49.6%), *P. aeruginosa* (21%), *K. pneumoniae* (18.6%) were predominant, followed by *S. aureus* (6.7*%)*,* E. coli* (3.9%) and *S. pneumoniae* (0.2%). Most isolates (94.4%) were identified from hospital-acquired cases. High prevalence of MDR and carbapenem resistance were identified in *A. baumannii* (96% and 95%), *P. aeruginosa* (56.7% and 57.1%), and *K. pneumoniae* (78% and 69.2%), respectively. Notably, resistance to ceftazidime-avibactam was detected in *K. pneumoniae* (34.3%), *P. aeruginosa* (29%), and *E. coli* (7.7%), while colistin resistance was found in *K. pneumoniae* (18.2%) and *A. baumannii* (2.8%). MRSA prevalence was 79.1%, though *S. aureus* remained susceptible to vancomycin, linezolid and ceftaroline. Most *bla*_NDM_-positive *K. pneumoniae* (62/71, 87.3%), *E. coli* (2/2, 100%), and *P. aeruginosa* (23/25, 85.2%) showed resistance to ceftazidime-avibactam. Whole genome sequencing revealed that the *bla*_NDM_-positive but ceftazidime-avibactam susceptible isolates (9 *K. pneumoniae* and 2 *P. aeruginosa*) carried truncated *bla*_NDM_. Overall, ceftazidime-avibactam was effective against *K. pneumoniae*, *E. coli*, and *P. aeruginosa* isolates carrying ESBL, ESBL and *bla*_OXA−48_, or ESBL and *bla*_KPC_. Alternatively, no detectable AMR genes were found in 35 ceftazidime-avibactam resistant *P. aeruginosa* isolates.

**Conclusions:**

Carbapenem-resistant Gram-negative pathogens were predominant among hospital-acquired LRTIs in Vietnam, with notable resistance to ceftazidime-avibactam and colistin. The lack of effective treatment for *A. baumannii* remains a major concern. We found a strong correlation between AMR phenotype and genotype among *K. pneumoniae* and *E. coli*, supporting gene-based therapy to guide ceftazidime-avibactam use. However, the presence of disrupted *bla*_NDM_ underscores the need to re-evaluate commercial PCR assays for carbapenemase detection.

**Supplementary Information:**

The online version contains supplementary material available at 10.1186/s12941-025-00818-3.

## Introduction

Lower respiratory tract infection (LRTI) represents a prominent global cause of mortality and morbidity, particularly among vulnerable populations such as children and elders [1, 2]. LRTI encompasses a diverse range of lung and airway infections beneath the vocal cords, each marked by distinctive epidemiological profiles, pathogeneses, clinical manifestations, and outcomes [3]. In 2019, an estimated 489 million LRTI cases were recorded worldwide, with nearly 2.5 million fatalities resulting from these infections [4]. These infections pose a higher mortality burden than other infectious diseases, including HIV/AIDS, malaria, and tuberculosis [5].

The etiology of LRTI is diverse and varies by geographic location, age, immune status, seasonality, and other factors like origin of infection (community- or hospital-acquired) [6, 7]. Community-acquired LRTI is defined as an infection with symptom onset before hospital admission or within 48 h of admission, typically caused by Gram-positive bacteria and respiratory viruses such as influenza and RSV [8, 9]. In contrast, hospital-acquired LRTI is defined as infection occurring 48 h or more after hospital admission, often caused by Gram-negative bacteria [9, 10]. Prevalent bacterial causative agents include Gram-positive bacteria such as *S. aureus* and *S. pneumoniae*, as well as Gram-negative bacteria (GNB) like *P. aeruginosa*, *K. pneumoniae*, *E. coli*, and *A. baumannii* [6, 11, 12]. There has been a surge in the prevalence of GNB as culprits of community-acquired pneumonia (CAP) and nosocomial pneumonia in the past decades [6, 13, 14]. The GNB prevalence in CAP ranges from under 5% in Europe to 20–30% in Asian countries like China, Japan, and Taiwan [15]. Meanwhile, nosocomial pneumonia GNB incidence exceeds 50% in many settings [13, 16]. GNB infections are often associated with poor outcomes due to inappropriate or delayed antimicrobial therapy [17].

The prevalence of multidrug-resistant (MDR) GNB and their antimicrobial resistance (AMR) profiles are geographically varied. While carbapenem-resistant *A. baumannii*, ESBL (extended-spectrum β-lactamase)-producing *K. pneumoniae* and *E. coli* are prevalent in the Asia-Pacific region, MDR *P. aeruginosa* are predominant in certain European regions, particularly Southern and Eastern Europe [18, 19]. Notably, the incidence of carbapenem-resistant Enterobacteriales (CRE) infections has increased in hospital settings throughout Asia [20], resulting in elevated mortality rates and increased healthcare expenditures [21]. ESBLs of type *bla*_CTX−M_ and carbapenemases such as *bla*_NDM_, *bla*_KPC_, and *bla*_OXA48_ have emerged in *E. coli* and *K. pneumoniae* lineages across Asia [22, 23]. The proliferation of ESBL and carbapenemases-producing GNB threatens the efficacy of empirical antimicrobial treatment for LRTI, leading to increased use of last-resort drugs such as ceftazidime-avibactam, colistin, tigecycline, and fosfomycin, which are expensive and potentially have more adverse effects.

Given the changing etiology and AMR profiles of LRTI, along with the introduction of new Beta-Lactam/Beta-Lactamase Inhibitors (BL/BLIs), such as ceftazidime-avibactam in Vietnam, ongoing surveillance is needed to improve treatment and infection control measures. Here, we conducted a multi-hospital survey to determine the microbiology and AMR landscape of LRTI across six major hospitals in Vietnam. We also present the first detailed analysis of the correlation between AMR phenotype and genotype for ceftazidime-avibactam.

## Materials and methods

### Study sites

This isolate-initiated survey was conducted between January 2022 and May 2023 on patients clinically diagnosed with LRTI across six major hospitals in Vietnam. These include Cho Ray Hospital, one of the largest general hospitals in Vietnam (level I) with 1200 beds, providing medical care for patients from Ho Chi Minh City (HCMC) as well as southern and central Vietnam, estimated to reach 57 million people (approximately 60% of the country’s total population of 95 million people).

The Hospital for Tropical Diseases is a large referral hospital for infectious diseases in southern Vietnam. Hospital for Tropical Diseases (HTD) has about 660 beds and serves more than 2500 outpatients per day. The majority of patients (70%) are residents of HCMC, while the remaining patients come from the surrounding provinces. Nhan Dan Gia Dinh General (NDGD) Hospital is a major tertiary teaching hospital with about 1500 beds, serving more than 4000 outpatients per day. The hospital provides medical care for residents of HCMC and patients from surrounding provinces.

Bach Mai Hospital in Ha Noi is the largest general hospital in Vietnam (level I). The hospital has more than 1900 beds and provides medical services for more than 900,000 outpatients and 100,000 inpatients every year. Viet-Duc Hospital is the largest surgical center in the North of Vietnam with more than 1500 beds. The hospital is also a national referral center for many types of patients at severe stages and nearly 70% of the patients are referred from the provincial hospitals. Every year, the hospital conducts more than 300,000 consultations and 60,000 operations. The National Hospital for Tropical Diseases is a 300-bed tertiary teaching hospital that provides medical care to patients with infectious diseases residing in Ha Noi and surrounding provinces. National Hospital for Tropical Diseases (NHTD) is also a referral hospital assigned to all provincial hospitals in the North of Vietnam for infectious diseases.

### Sample collection

A total of 1000 non-duplicate isolates were obtained from routine microbiological cultures of respiratory samples collected from hospitalized patients clinically diagnosed with LRTI during 2022–2023. The target bacterial pathogens were selected based on previous studies of LRTI in Vietnam and SEA [14, 15, 24–26], including *A. baumannii*, *P. aeruginosa*, *K. pneumoniae*, *E. coli*, *S. aureus*, and *S. pneumoniae.* An episode of LRTI in this study was defined as isolation of at least one target pathogen from one of the respiratory specimens (sputum, bronchoalveolar lavage fluid, bronchial washing, pleural aspirate, endotracheal aspirate, protected specimen brush, lung biopsy), collected from a patient with a clinical syndrome indicative of LRTI, as determined by the attending physician.

### Bacterial culture and identification

All target bacterial isolates were obtained through routine microbiological cultures at six collaborating hospitals, following the National Technical Guidelines on Clinical Microbiology Diagnostics issued by Vietnam’s Ministry of Health under Decision No.1539/QD-BYT [27]. Sputum and tracheal aspirate (TA) samples were collected into a sterile container and initially assessed by Gram staining and examination under direct microscopy. Samples with fewer than 10 epithelial cells and more than 25 leukocytes in each area upon 100 × magnification were considered good quality for bacterial culture [28]. Sputum samples were processed using a quadrant streaking technique. TA samples were first liquefied with Sputasol liquid (Oxoid, USA) with a ratio of 1:1 and diluted with MRD (maximum recovery diluent) broth with ratio of 1:9 before microbiological testing. Subsequently, 1 µl of suspension inoculated into blood agar, MacConkey agar and chocolate agar media (Oxoid, USA), and incubated for 24–48 h at 35–37 °C or at 35–37 °C with 5% CO2. TA cultures were considered positive when known respiratory pathogens were isolated at concentrations ≥ 10⁵ CFU/mL. Bronchoalveolar lavage (BAL) samples were cultured directly onto agar plates, and a threshold of ≥ 10⁴ CFU/mL was used to define the presence of respiratory pathogens.

Bacterial identification was performed using the automated VITEK-2 system (Biomérieux) or the Phoenix M50 system (Becton Dickinson) in the hospital microbiology laboratories. Subsequently, the bacterial isolates were transferred to the Oxford University Clinical Research Unit (OUCRU) laboratory, where MALDI-TOF (Bruker, Germany) or in-house real-time PCR was used to reconfirm the species when needed [29]. The results were determined based on the findings from OUCRU lab in case of discrepancies.

### Antimicrobial susceptibility testing (AST)

AST was conducted on bacterial isolates using the automated VITEK-2, the BD Phoenix system, following the routine procedures at the participating hospitals. For Gram-negative bacteria, antimicrobials including ciprofloxacin, levofloxacin, gentamicin, amikacin, piperacillin, piperacillin/tazobactam, cefepime, ceftriaxone, ceftazidime, ampicillin, trimethoprim-sulfamethoxazole, imipenem, meropenem were commonly tested in hospital microbiology labs. For Gram-positive bacteria, antimicrobials such as ciprofloxacin, erythromycin, oxacillin, vancomycin, daptomycin, linezolid, tigecycline, teicoplanin, clindamycin and trimethoprim-sulfamethoxazole, were included for AST.

Furthermore, the minimum inhibitory concentrations (MICs) of selective antimicrobial agents were determined by E-test for Gram-negative bacteria (ceftazidime-avibactam, colistin) (bioMérieux) and Gram-positive bacteria (ceftaroline) (bioMérieux). Colistin susceptibility of *A. baumannii* was assessed using the broth microdilution method. The AST results were interpreted according to the Clinical and Laboratory Standards Institute (CLSI) 2022 guidelines [30].

Daily internal quality control was conducted using suitable ATCC control strains such as *E. coli* ATCC25922, *P. aeruginosa* ATCC27853, *K. pneumoniae* ATCC700603, *S. aureus* ATCC25923, following the CLSI guidelines and manufacturers’ protocols.

### Data collection

Where accessible, patient data was collected from the electronic medical records at the participating hospitals. This dataset included the following details: date of admission, age, gender, place of admission (home/hospital transfer), underlying conditions, date of ICU admission, date of hospital discharge, and discharge outcome diagnosis. Microbiological data was collected from the hospital microbiology labs, including sample type, dates of sample collection and positive culture, ward of collection, clinical diagnosis, and AST results. A case report form was designed to collect these data. After de-identification, the information was securely stored in an electronic Clires database at OUCRU with restricted access.

The clinical outcome was classified as good (discharged with recovery), poor (death or discharged home to die), or unknown (patient transferred to another hospital or self-discharged). Hospital-acquired infection was a positive culture with at least one clinically relevant target pathogen from a patient with clinically suspected LRTI beyond the initial 48 h of admission. Community-acquired infection was defined as a positive culture with at least one clinically relevant target pathogen from a patient with clinically suspected LRTI within 48 h of hospital admission and without a history of hospital transfer. MDR was defined as acquired non-susceptibility to at least one agent in three or more antimicrobial categories [30]. The antimicrobial groups for Gram-negative bacteria included aminoglycosides, penicillins with beta-lactamase inhibitors, 3rd/4th generation cephalosporins, carbapenems, fluoroquinolones, folate pathway inhibitors, polymyxins, and glycylcycline. For Gram-positive bacteria, antimicrobial classes included aminoglycosides, rifamycins, anti-staphylococcal B-lactams (or cephamycins), fluoroquinolones, folate pathway inhibitors, fucidanes, glycopeptides, lincosamides, macrolides, oxazolidinones, and tetracyclines.

### PCR detection of commonly acquired AMR genes

The identification of prevalent AMR genes conferring resistance to 3rd/4th generation cephalosporins (ESBL genes including *bla*_TEM_, *bla*_SHV_, *bla*_CTX−M−1_, *bla*_CTX−M−9_) and carbapenems (*bla*_NDM_, *bla*_OXA−23_, *bla*_OXA−48_, *bla*_IMP_, *bla*_KPC_), vancomycin (*vanA*, *vanB*) was performed using our in-house multiplex PCR assays [29]. PCR amplifications were performed using SensiFASTTM HRM Kit (Bioline, USA) on a LightCycler 480II instrument (Roche Applied Sciences, UK) under the following thermal conditions: initial denaturation at 95 °C for 5 min, followed by 40 cycles of denaturation at 95 °C for 10 s, annealing at 60 °C for 30 s, and extension at 72 °C for 30 s. Subsequently, a melting curve analysis (MCA) was conducted on the same thermocycler, ranging from 65 to 95 °C, followed by a cooling cycle at 37 °C for 30 s. Fluorescence was continuously monitored, and the melting temperature (Tm) was calculated by plotting the negative derivative of fluorescence over temperature versus temperature (-dF/dT versus T). These assays were directly applied to bacterial colonies. These AMR genes are commonly found among bacterial pathogens responsible for LRTI in Vietnam and other regions [31, 32]. The primer sequences are previously described [29].

### Whole genome sequencing (WGS)

WGS was performed on 5 isolates (4 *K. pneumoniae* and 1 *P. aeruginosa*) that were PCR-positive with *bla*_NDM_ but phenotypically susceptible to ceftazidime-avibactam. The genomic DNA of selected isolates was extracted using the Wizard Genomic DNA Extraction Kit (Promega, USA), and Nextera XT kit was used to prepare a library from 1 ng of genomic DNA. WGS was performed on an Illumina HiSeq platform (Illumina, USA) to generate 150 bp paired-end reads. Raw Illumina reads were subjected to quality control using FastQC v0.11.5 [33]. The de novo assembly of these reads was conducted with Unicycler v0.4.8b, using default parameters [34]. The resulting assembled sequences were annotated through Prokka v1.14.5 [35]. To identify antimicrobial resistance (AMR) genes, we employed SRST2 v0.2.0 with the ARG-ANNOT antimicrobial resistance database [36]. For the pairwise alignment of the detected *bla*_NDM_, we used the Basic Local Alignment Search Tool (BLASTn) with the default parameters [37]. The comparative genomics of the *bla*_NDM_ gene were performed using the web application pyGenomeViz [38].

### Statistical analysis

Mathematical calculations were used to determine the MIC_50_ and MIC_90_ values, which represent the MICs required to inhibit the growth of 50% and 90% of bacteria, respectively. Additional measures of MIC variability, including the MIC range and mode (the most frequent MIC value), were also calculated. The Skewness score was used to test whether the MIC data was normally distributed.

Statistical analyses were conducted utilizing the R software (version 4.3.2). The mean (standard deviation, SD) or median (interquartile range, IQR) was reported for continuous variables. As appropriate, group comparisons were conducted using either an unpaired samples t-test or a Wilcoxon rank sum test. Categorical variables were characterized using frequencies and percentages, and group comparisons were conducted using either a chi-square test or, where applicable, Fisher’s exact test. Statistically significant results were defined as those with p values ≤ 0.05.

## Rsults

### Demographic characteristics of LRTI patients

The median age of all LRTI patients was 63 years old (IQR: 49–74 years) (Table 1). Generally, male patients accounted for a higher proportion of LRTI cases than females (65.7% vs. 34.3%, *p* < 0.001). Among the 1,000 LRTI bacteria identified, 56.2% were from ICU wards, and 43.8% were from non-ICU wards. The median length of stay was 20 days (IQR: 12–33 days) and the majority of LRTI cases (94.4%, 626/663) were hospital acquired. Out of LRTI patients with available outcome data, 37.1% (276/743) died in the hospital or were discharged to die at home, 35.4% (263/743) were discharged with incomplete recovery, 7.5% (56/743) were discharged with complete recovery, and 19.9% (148/743) were transferred to another hospital for further care.

**Table 1:** General characteristics of lower respiratory tract infections patients

Characteristic	Total = 1000*
Age (years)	63.0 (49.0, 74.0)
Sex	
Female	343.0 (34.3%)
Male	657.0 (65.7%)
Length of stay (days)	20.0 (12.0, 33.0)
Missing	250
Outcome	
Death or discharge to die	276.0 (37.1%)
Discharged with a complete recovery	56.0 (7.5%)
Discharged with an incomplete recovery	263.0 (35.4%)
Transferred to another hospital	148.0 (19.9%)
Missing	257

* Median (Q1, Q3); n (%)

## Distribution of LRTI pathogens

Among 1000 bacterial isolates collected from January 2022 to May 2023, 931 (93.1%) were Gram-negative bacteria, while the remaining 69 (6.9%) were Gram-positive. The predominant Gram-negative bacteria included *A. baumannii* (49.6%), *P. aeruginosa* (21%), and *K. pneumoniae* (18.6%), while *E. coli* only constituted 3.9%. Among Gram-positive bacteria, *S. aureus* accounted for 6.7% of all, whereas *S. pneumoniae* was comparatively less frequent, comprising only 0.2% of total isolates.

**Fig. 1:**
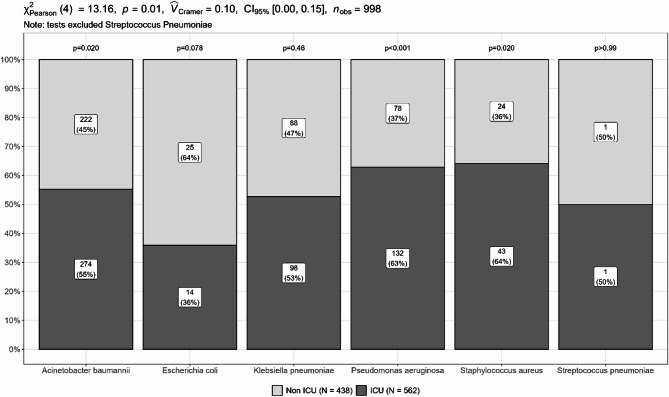
Distribution of 6 target LRTI pathogens between ICU and non-ICU sources

The occurrence of the six pathogens differed significantly in ICUs versus non-ICU wards (*p* < 0.05) (Fig. 1). *A. baumannii*, *K. pneumoniae*, *P. aeruginosa*, and *S. aureus* were commonly found in ICUs, while *E. coli* was more frequently found in non-ICU wards. Most target pathogens were identified in hospital-acquired LRTI patients with prolonged hospital stays, including *A. baumannii* (95.3%, 326/342), *P. aeruginosa* (95.1%, 137/144), *K. pneumoniae* (91.5%, 107/117), *E. coli* (80%, 16/20) and *S. aureus* (95.2%, 40/42).

### Antimicrobial resistance profiles of LRTI bacteria

*A. baumannii* exhibited high proportions of resistance to the commonly used antibiotics, including 94.8% (470/496) and 95.9% (441/460) for ceftazidime and cefepime, 88.3% (438/496) and 92.6% (426/460) for ciprofloxacin and levofloxacin, 80.4% (397/494) for gentamicin and 87.5% (434/496) for piperacillin-tazobactam (Table 2). The resistance prevalence to imipenem and meropenem was 95.5% (471/493) and 95.4% (461/483), respectively, while colistin resistance was 2.8% (14/496). The resistance proportion of *A. baumannii* isolates tested against ceftazidime-avibactam was 96.8% (149.0/154.0). Overall, 96.0% (476/496) of *A. baumannii* isolates were MDR (Table 2), and 2.8% (14/496) were PDR.

**Table 2:** Antimicrobial resistance patterns of Gram-negative LRTI pathogens

	*A. baumanni*i	*E. coli*	*K. pneumoniae*	*P. aeruginosa*
	*N* = 496*	*N* = 39*	*N* = 186*	*N* = 210*
MDR	96.0% (476.0/496.0)	84.6% (33.0/39.0)	78.0% (145.0/186.0)	56.7% (119.0/210.0)
Cephalosporins (4th gen.)				
Cefepime	95.9% (441.0/460.0)	50.0% (10.0/20.0)	73.2% (90.0/123.0)	47.8% (76.0/159.0)
Cephalosporins (3rd gen.)				
Ceftazidime/avibactam	96.8% (149.0/154.0)	7.7% (3.0/39.0)	34.4% (64.0/186.0)	29.0% (61.0/210.0)
Ceftazidime	94.8% (470.0/496.0)	62.9% (22.0/35.0)	73.7% (126.0/171.0)	49.5% (103.0/208.0)
Carbapenems				
Meropenem	95.4% (461.0/483.0)	7.7% (2.0/26.0)	69.8% (113.0/162.0)	56.5% (105.0/186.0)
Imipenem	95.5% (471.0/493.0)	10.3% (4.0/39.0)	64.3% (119.0/185.0)	56.7% (115.0/203.0)
Ertapenem	–	23.1% (9.0/39.0)	65.8% (106.0/161.0)	50.0% (1.0/2.0)
Polymyxins				
Colistin	2.8% (14.0/496.0)	0.0% (0.0/32.0)	18.1% (25.0/138.0)	0.6% (1.0/156.0)
Aminoglycosides				
Amikacin	84.4% (238.0/282.0)	2.6% (1.0/39.0)	30.6% (57.0/186.0)	44.0% (84.0/191.0)
Gentamicin	80.4% (397.0/494.0)	46.2% (18.0/39.0)	42.7% (79.0/185.0)	51.2% (105.0/205.0)
Beta-lactams/penicillins				
Piperacillin/tazobactam	87.5% (434.0/496.0)	34.2% (13.0/38.0)	73.1% (136.0/186.0)	48.2% (96.0/199.0)
Quinolones				
Ciprofloxacin	88.3% (438.0/496.0)	79.5% (31.0/39.0)	81.1% (150.0/185.0)	58.5% (121.0/207.0)
Levofloxacin	92.6% (426.0/460.0)	75.0% (21.0/28.0)	76.1% (108.0/142.0)	60.6% (97.0/160.0)
Trimethoprims				
Trimethoprim/sulfamethoxazole	70.2% (344.0/490.0)	66.7% (26.0/39.0)	51.7% (93.0/180.0)	33.3% (1.0/3.0)

* % Resistant, (Resistant case/Total)

Among the tested *P. aeruginosa* isolates, resistance proportions were 48.2% (96/199) to piperacillin-tazobactam, 49.5% (103/208) to ceftazidime, 58.5% (121/207) to ciprofloxacin, 56.7% (115/203) to imipenem, 56.5% (105/186) to meropenem, and 51.2% (105/205) to gentamicin. Of total isolates, 29% (61/210) were resistant to ceftazidime-avibactam. The percentage of MDR *P. aeruginosa* was 56.7% (119.0/210.0).

Among the tested *K. pneumoniae* isolates, a high resistance prevalence was observed for ceftazidime (73.7%, 126/171), cefepime (73.2%, 90/123), ciprofloxacin (81.1%, 150/185), levofloxacin (76.1%, 108/142), gentamicin (42.7%, 79/185), piperacillin-tazobactam (73.1%, 136/186), imipenem (64.3%, 119/185) and meropenem (69.8%, 113/162). Concerningly, 34.4% of *K. pneumoniae* isolates displayed resistance to ceftazidime-avibactam, and 18.1% were resistant to colistin. The proportion of MDR *K. pneumoniae* was 78% (145/186).

Among the tested *E. coli* isolates, 62.9% (22/35), 50% (10/20), 79.5% (31/39), 75% (21/28), 46.2% (18/39), and 34.2% (13/38) were resistant to ceftazidime, cefepime, ciprofloxacin, levofloxacin, gentamicin and piperacillin-tazobactam, respectively. The proportions of *E. coli* resistant to imipenem, meropenem, and ceftazidime-avibactam were 10.3% (4/39), 7.7% (2/26), and 7.7% (3/39), respectively. None of the *E. coli* isolates were resistant to colistin. The MDR *E. coli* was 84.6% (33/39).

Among the Gram-positives, the resistance proportions of *S. aureus* to oxacillin, ciprofloxacin, moxifloxacin, trimethoprim-sulfamethoxazole, clindamycin, and erythromycin were 74.6% (50/67), 37.2% (16/43), 34.1% (14/41), 17.8% (8/45), 66% (31/47), and 70.2% (33/47), respectively. All *S. aureus* isolates were susceptible to vancomycin, teicoplanin, and ceftaroline, and 97.7% (42/43) were susceptible to linezolid. MRSA accounted for 79.1% (53/67) of *S. aureus* isolates (Table 3).

**Table 3:** Antimicrobial resistance patterns of Gram-positive LRTI pathogens

	*S. aureus*	*S. pneumoniae*
	*N* = 67*	*N* = 2*
MDR	79.1% (53.0/67.0)	0.0% (0.0/2.0)
Cephalosporins (5th gen.)		
Ceftaroline	0.0% (0.0/67.0)	0.0% (0.0/2.0)
Beta-lactams/penicillins		
Oxacillin	74.6% (50.0/67.0)	–
Glycopeptides		
Vancomycin	0% (0.0/43.0)	0.0% (0.0/2.0)
Teicoplanin	0.0% (0.0/41.0)	–
Macrolides/lincosamides		
Clindamycin	66.0% (31.0/47.0)	100.0% (2.0/2.0)
Erythromycin	70.2% (33.0/47.0)	100.0% (2.0/2.0)
Oxazolidinones		
Linezolid	2.3% (1.0/43.0)	0.0% (0.0/2.0)
Quinolones		
Ciprofloxacin	37.2% (16.0/43.0)	–
Moxifloxacin	34.1% (14.0/41.0)	0.0% (0.0/2.0)
Levofloxacin	0.0% (0.0/2.0)	0.0% (0.0/2.0)
Tetracyclines		
Doxycycline	18.6% (8.0/43.0)	–
Trimethoprims		
Trimethoprim/sulfamethoxazole	17.8% (8.0/45.0)	100.0% (2.0/2.0)

* % Resistant, (Resistant cases/Total)

### In vitro activity of ceftazidime-avibactam and other comparator agents

Overall, among the 186 *K. pneumoniae* and 39 *E. coli*, 65.6% and 89.7%, respectively, were susceptible to ceftazidime-avibactam, with MIC values ≤ 3 mg/L (MIC_50_, 1.5 mg/L) (Table 4, Supplementary Figs. 2, 3). For *P. aeruginosa* isolates, the susceptibility percentage was 69% (145/210), with MIC values ≤ 8 mg/L (MIC_50_, 2 mg/L) (Table 4, Supplementary Fig. 4). *K. pneumoniae*, *E. coli*, and *P. aeruginosa* isolates were significantly more susceptible to ceftazidime-avibactam than to ceftazidime, imipenem and meropenem. *E. coli* (100%, 32/32) and *P. aeruginosa* (99.4%, 155/156) isolates remained susceptible to colistin, however, the susceptible proportion was lower in *K. pneumoniae* (81.2%, 112/138) (Table 4). The MIC_50_ and MIC_90_ values of colistin were 0.19 mg/L and 8 mg/L for *K. pneumoniae* (*n* = 138), 0.75 mg/L and 1.5 mg/L for *P. aeruginosa* (*n* = 156), and 0.125 mg/L and 0.19 mg/L for *E. coli* isolates (*n* = 32), respectively.

**Table 4:** In vitro activity of ceftazidime-avibactam and comparators against Gram-negative bacteria

	Interpretation (%)		MIC (mg/l)					
	*N*	Susceptible	*N*	MIC Range^1^	MIC50	MIC90	MIC Mode^2^	Skewness Score
*Acinetobacter baumannii*								
Colistin	496	482 (97.2%)	496	< 0.062 to 32	0.5	1	0.5 (219/496)	7.76
Ceftazidime	496	24 (4.8%)	325	2 to ≥ 64	≥ 64	≥ 64	≥ 64 (308/325)	−4.2
Imipenem	493	21 (4.3%)	327	≤ 0.25 to ≥ 16	≥ 16	≥ 16	≥ 16 (311/327)	−4.7
Meropenem	483	22 (4.6%)	261	≤ 0.25 to ≥ 16	≥ 16	≥ 16	≥ 16 (249/261)	−4.8
*Escherichia coli*								
Colistin	32	32 (100%)	32	0.064 to 2	0.125	0.19	0.125 (18/32)	5.01
Ceftazidime	35	12 (34.3%)	26	≤ 1 to ≥ 64	16	≥ 64	≥ 64 (12/26)	0.07
Imipenem	39	29 (74.4%)	23	≤ 0.25 to ≥ 16	≤ 0.25	2	≤ 0.25 (18/23)	4.31
Meropenem	26	22 (84.6%)	13	≤ 0.25 to ≥ 16	≤ 0.25	1	≤ 0.25 (10/13)	3.15
Ceftazidime/Avibactam	39	35 (89.7%)	39	0.047 to ≥ 256	0.19	12	0.19 (9/39)	3.26
*Klebsiella pneumoniae*								
Colistin	138	112 (81.2%)	138	0.094 to 96	0.19	8	0.125 (57/138)	4.06
Ceftazidime	171	44 (25.7%)	119	≤ 0.5 to ≥ 64	≥ 64	≥ 64	≥ 64 (83/119)	−0.9
Imipenem	185	58 (31.4%)	106	≤ 0.25 to ≥ 16	≥ 16	≥ 16	≥ 16 (57/106)	−0.22
Meropenem	162	47 (29%)	92	≤ 0.25 to ≥ 16	≥ 16	≥ 16	≥ 16 (61/87)	−0.92
Ceftazidime/Avibactam	186	122 (65.6%)	186	0.016 to ≥ 256	1.5	≥ 256	≥ 256 (58/186)	0.69
*Pseudomonas aeruginosa*								
Colistin	156	155 (99.4%)	156	0.064 to 4	0.75	1.5	1 (55/156)	2.3
Ceftazidime	208	99 (47.6%)	68	≤ 1 to ≥ 64	16	≥ 64	≥ 64 (32/68)	0.05
Imipenem	203	87 (42.9%)	73	1 to ≥ 16	≥ 16	≥ 16	≥ 16 (39/73)	−0.2
Meropenem	186	77 (41.4%)	75	≤ 0.25 to ≥ 16	≥ 16	≥ 16	≥ 16 (38/73)	−0.12
Ceftazidime/Avibactam	210	145 (69%)	210	0.25 to ≥ 256	2	≥ 256	≥ 256 (53/210)	1.04

^1^ Min; Max

^2^ MIC mode (Number of cases/Total cases)

Notably, the carbapenem-resistant (CR) isolates of *K. pneumoniae* (*n* = 121), *P. aeruginosa* (*n* = 119), and *E. coli* (*n* = 2) demonstrated reduced susceptibility to ceftazidime-avibactam compared to non-CR isolates (Table S1). Among the tested isolates, only 47.1% (*n* = 57) of CR *K. pneumoniae* and 49.6% (*n* = 59) of CR *P. aeruginosa* were susceptible to ceftazidime-avibactam.

### In vitro activity of ceftaroline and other comparators against ***S. aureus***

All tested *S. aureus* isolates displayed susceptibility to ceftaroline with an MIC_90_ of 0.5 mg/L (Table 5). Ceftaroline exhibited efficacy against methicillin-susceptible *S. aureus* (MSSA) and MRSA with a MIC_90_ of ≤ 0.5 mg/L. In comparison, the three major treatment options for MRSA, vancomycin, teicoplanin, and linezolid, demonstrated efficacy against MRSA, with an MIC_90_ of 1 mg/L, ≤ 0.5 mg/L, and 2 mg/L, respectively (Table 5).

**Table 5:** In vitro activity of Ceftaroline and comparators against *S. aureus* isolates

	Interpretation (%)					MIC (mg/l)					
	N	Sensitive	Intermediate	Resistance	N		MIC range^1^	MIC50	MIC90	MIC Mode^2^	Skewness score
*MRSA*											
Linezolid	35	35 (100%)	0 (0%)	0 (0%)	17		1 to 4	2	2	2 (15/17)	2.3
Teicoplanin	33	33 (100%)	0 (0%)	0 (0%)	15		≤ 0.5 to 4	≤ 0.5	≤ 0.5	≤ 0.5 (14/15)	3.47
Vancomycin	35	35 (100%)	0 (0%)	0 (0%)	12		≤ 0.5 to 2	1	1	1 (6/12)	1.37
Ceftaroline	52	52 (100%)	0 (0%)	0 (0%)	52		0.19 to 1	0.38	0.5	0.38 (32/52)	2.41
*MSSA*											
Linezolid	8	7 (87.5%)	0 (0%)	1 (12.5%)	2		2 to 2	2	2	2 (2/2)	
Teicoplanin	8	8 (100%)	0 (0%)	0 (0%)	2		≤ 0.5 to ≤ 0.5	≤ 0.5	≤ 0.5	≤ 0.5 (2/2)	
Vancomycin	8	8 (100%)	0 (0%)	0 (0%)	2		1 to 1	1	1	1 (2/2)	
Ceftaroline	15	15 (100%)	0 (0%)	0 (0%)	15		0.094 to 0.38	0.19	0.25	0.19 (8/15)	1.03

^1^Min; Max

^2^MIC mode (number of cases/total cases)

### AMR gene profiles and their correlation with AMR phenotypes

The presence of commonly acquired AMR genes identified by PCR assays in each bacterium is summarized in Table 6. Notably, ESBL-encoding *bla*_CTX−M−1_/*bla*_CTX−M−9_ were frequently identified in *E. coli* (69.2%, 27/39) and *K. pneumoniae* (66.1%,123/186). In contrast, these genes were only found in 5.3% (11/210) of *P. aeruginosa* and 2% (10/496) of *A. baumannii* isolates. As expected, most *A. baumannii* isolates carried the carbapenemase gene *bla*_OXA−23_ (86.1%, 427/496); however, this gene was also detected in 2.7% (5/186) of *K. pneumoniae* and 0.5% (1/210) of *P. aeruginosa* isolates. A high prevalence of *bla*_OXA−48_, *bla*_NDM_, and *bla*_KPC_ was found in *K. pneumoniae* isolates with respective proportions of 39.2% (73/186), 38.2% (71/186), and 32.8% (61/186). *Bla*_OXA−48_ and *bla*_NDM_ genes were also found in *P. aeruginosa* (1.9% and 11.9%), *E. coli* (10.3% and 5.1%), and *A. baumannii* (3.6% and 4.8%) isolates, although with lower frequencies. In *S. aureus*, the *mecA* genes were identified in 77.6% (52/67) of isolates (Table 6).

**Table 6:** PCR-based detection of AMR genes in LRTI pathogens

Gram-negative bacteria					
AMR genes	Overall, *N* = 931*	*A. baumannii*, *N* = 496*	*E. coli*, *N* = 39*	*K. pneumoniae*, *N* = 186*	*P. aeruginosa*, *N* = 210*
*bla*_CTX−M−1_	132 (14.2%)	4 (0.8%)	16 (41%)	102 (54.8%)	10 (4.8%)
*bla*_CTX−M−9_	39 (4.2%)	6 (1.2%)	11 (28.2%)	21 (11.3%)	1 (0.5%)
*bla*_TEM_	508 (54.6%)	353 (71.2%)	17 (43.6%)	127 (68.3%)	11 (5.2%)
*bla*_SHV_	164 (17.6%)	1 (0.2%)	0 (0%)	150 (80.6%)	13 (6.2%)
*bla*_OXA−23_	433 (46.5%)	427 (86.1%)	0 (0%)	5 (2.7%)	1 (0.5%)
*bla*_OXA−48_	99 (10.6%)	18 (3.6%)	4 (10.3%)	73 (39.2%)	4 (1.9%)
*bla*_NDM_	122 (13.1%)	24 (4.8%)	2 (5.1%)	71 (38.2%)	25 (11.9%)
*bla*_IMP_	6 (0.6%)	4 (0.8%)	0 (0%)	0 (0%)	2 (1.0%)
*bla*_IMP_	62 (6.7%)	0 (0%)	1 (2.6%)	61 (32.8%)	0 (0%)

Gram-positive bacteria					
AMR genes	Overall, N = 69*	*Staphylococcus aureus*, N = 67*	*Streptococcus pneumoniae*, N = 2*		
*mecA*	53 (76.8%)	52 (77.6%)	1 (50%)		
*vanA*	0 (0%)	0 (0%)	0 (0%)		
*vanB*	0 (0%)	0 (0%)	0 (0%)		

* n (%)

**Fig. 2:**
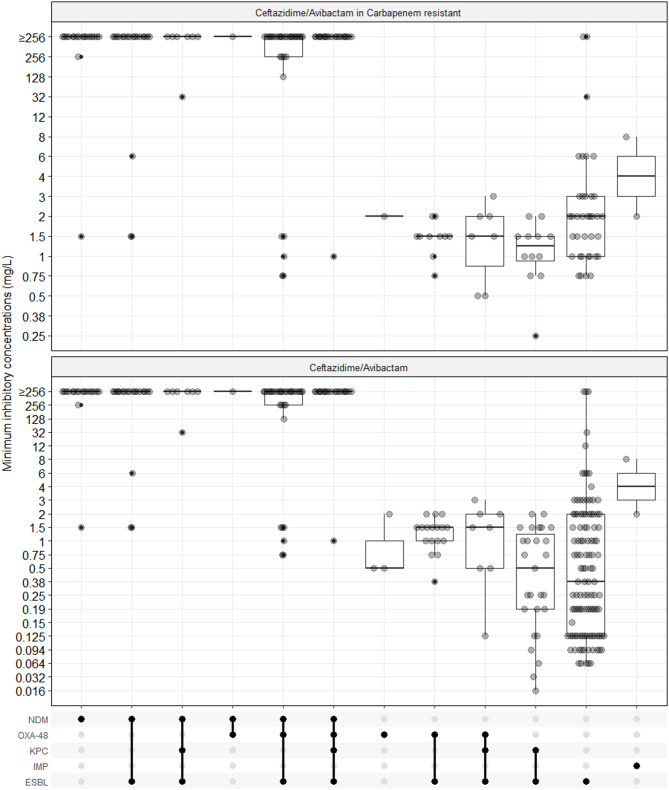
Distribution of ceftazidime-avibactam MICs by presence of AMR genes in total (below graph) and carbapenem-resistant isolates of *E. coli*, *K. pneumoniae*, and *P. aeruginosa* (above graph)

Among 64 ceftazidime-avibactam resistant *K. pneumoniae* isolates, various AMR gene patterns involving *bla*_NDM_ were identified, each associated with an MIC_50_ and MIC_90_ value of ≥ 256 mg/L (Table 7; Fig. 2). Specifically, 11 isolates (17.2%) carried both *bla*_NDM_ and ESBL gene, 51 isolates (79.7%) harboured *bla*_NDM_, ESBL gene, along with either *bla*_OXA−48_ or *bla*_KPC_. Among 61 ceftazidime-avibactam resistant *P. aeruginosa* isolates, 23 (37.7%) carried *bla*_NDM_, with or without *bla*_OXA−48_ and ESBL gene. No detectable AMR genes were found in 35 isolates (57.3%), and the remaining 3 isolates (4.9%) harbored only the ESBL gene. Out of 3 ceftazidime-avibactam resistant *E. coli* isolates, one carried *bla*_NDM_ and ESBL gene; another harbored *bla*_NDM_, *bla*_OXA−48_, *bla*_KPC_ and ESBL gene, while the third had no detectable AMR genes (Table 7).

**Table 7:** Correlation between PCR-detected AMR genes and ceftazidime-avibactam mics in *E. coli*, *K. pneumoniae*, and *P. aeruginosa* isolates

	PCR-positive isolates^1^	cef-avi MIC Range (PCR-positive isolates)	cef-avi MIC50	cef-avi MIC90	No. cef-avi resistant isolates
*Escherichia coli* (*N* = 39)					
ESBL	30 (76.9%)	0.064 to 12	0.19	0.75	0
NDM + ESBL	1 (2.6%)	≥ 256 to ≥ 256	≥ 256	≥ 256	1
NDM + OXA-48 + KPC + ESBL	1 (2.6%)	≥ 256 to ≥ 256	≥ 256	≥ 256	1
OXA-48 + ESBL	3 (7.7%)	0.38 to 2	1.5	2	0
None	4 (10.3%)	0.047 to 192	0.19	0.19	1
*Klebsiella pneumoniae* (*N* = 186)					
ESBL	58 (31.2%)	0.064 to ≥ 256	0.25	1.5	1
KPC + ESBL	27 (14.5%)	0.016 to 2	0.5	1.5	0
NDM + ESBL	13 (7.0%)	1.5 to ≥ 256	≥ 256	≥ 256	11
NDM + KPC + ESBL	7 (3.8%)	32 to ≥ 256	≥ 256	≥ 256	7
NDM + OXA-48 + ESBL	32 (17.2%)	0.75 to ≥ 256	≥ 256	≥ 256	26
NDM + OXA-48 + KPC + ESBL	19 (10.2%)	1 to ≥ 256	≥ 256	≥ 256	18
OXA-48	1 (0.5%)	0.5 to 0.5	0.5	0.5	0
OXA-48 + ESBL	13 (7%)	0.75 to 2	1.5	1.5	0
OXA-48 + KPC + ESBL	8 (4.3%)	0.125 to 3	1.5	2	0
None	8 (4.3%)	0.064 to ≥ 256	0.125	1	1
*Pseudomonas aeruginosa* (*N* = 210)					
ESBL	24 (11.4%)	1 to ≥ 256	3	6	3
IMP	2 (1%)	2 to 8	2	2	0
NDM	18 (8.6%)	1.5 to ≥ 256	≥ 256	≥ 256	17
NDM + ESBL	6 (2.9%)	6 to ≥ 256	≥ 256	≥ 256	5
NDM + OXA-48	1 (0.5%)	≥ 256 to ≥ 256	≥ 256	≥ 256	1
OXA-48	2 (1%)	0.5 to 2	0.5	0.5	0
OXA-48 + ESBL	1 (0.5%)	2 to 2	2	2	0
None	156 (74.3%)	0.25 to ≥ 256	1.5	≥ 256	35

^*1*^% (N/total)

Notably, 9 *K. pneumoniae* and 2 *P. aeruginosa* isolates were PCR-positive for *bla*_NDM_ but remained susceptible to ceftazidime-avibactam. WGS analysis of selective isolates found that these *K. pneumoniae* isolates harbored *bla*_OXA−48_ and a truncated *bla*_NDM_ gene, missing the nucleotide region spanning positions 10–300 bp. Similarly, in the *P. aeruginosa* isolate, the *bla*_NDM_ was truncated, lacking nucleotides from positions + 794 to 813 bp.

## Discussion

Our multi-hospital surveillance effort fills the knowledge gaps in the microbiology and AMR profile of LRTI in Vietnam, particularly given the lack of susceptibility data of novel BL/BLIs such as ceftazidime-avibactam. Our study highlights the significant problem of Gram-negative pathogens, including *A. baumannii*, *P. aeruginosa*, and *K. pneumoniae*, as the most frequent causes of LRTI, concurring with finding from previous studies [29, 39–42]. Notably, these significant pathogens exhibited an extremely high level of MDR and resistance to treatment drugs, spanning both ICUs and non-ICU wards. Although existing data are limited, our findings indicate a rising resistance level compared to previous studies conducted in Vietnam [25, 43, 44]. As evidenced here, *A. baumannii* isolates were extremely drug-resistant and only susceptible to colistin. Furthermore, a high prevalence of MDR *K. pneumoniae* and MDR *P. aeruginosa* was found and their rising resistance to treatment drugs such as carbapenems, colistin, and ceftazidime-avibactam severely limits treatment options. Notably, high levels of carbapenem resistance were detected in *K. pneumoniae*, *P. aeruginosa*, and *A. baumannii* at 95.0%, 57.1%, and 69.2%, respectively. These proportions are markedly higher than those reported in the 2016–2017 VINARES national surveillance, which were 27%, 45%, and 79%, respectively [45] These proportions also exceed those reported for LRTIs in other Asian countries [46] and globally [47]. In this study, the novel BL/BLI, ceftazidime-avibactam, approved in Vietnam back in 2020, exhibited lower susceptibility levels compared to data from other multi-country surveillance studies on ceftazidime-avibactam against Enterobacterales and *P. aeruginosa* [48, 49]. There might be several reasons for this, including a delayed importation of ceftazidime-avibactam coupled with the heavy reliance on carbapenem use, and the widespread circulation of carbapenem-resistant GNB amid the COVID-19 pandemic. To navigate these challenges, it is crucial to follow the antibiotic development pipeline and expedite the introduction of new antibiotics into Vietnam. For example, novel beta-lactam/beta-lactamase inhibitor combinations recently approved by FDA, such as cefepime-enmetazobactam [50] and those that have completed Phase 3 clinical trials, including aztreonam-avibactam (Pfizer) [51] and cefepime-taniborbactam (Venatorx Pharmaceuticals) [52]or are currently in Phase 3 trials, such as cefepime/zidebactam (Clinicaltrials.gov Identifier. NCT04979806, Wockhardt) [53], have shown promise for the treatment of severe infections caused by MDR/CR Gram-negative bacteria. Furthermore, molecular surveillance has proven essential for tracking trends in epidemiology, etiology, and antimicrobial resistance, and should be further reinforced within the local hospital settings.

Our findings revealed the commonplace of *bla*_NDM_ carbapenemases across different Gram-negative bacteria, particularly the occurrence of *bla*_NDM_ and *bla*_OXA−48_ combination in *K. pneumoniae* which has been increasingly reported [54]. Further investigation into the genetic background of these isolates is needed to determine whether this rise is driven by clonal spread or horizontal plasmid transmission. Overall, our study showed a strong correlation between AMR phenotype and the genotype for ceftazidime-avibactam resistance. Ceftazidime–avibactam is ineffective against *A. baumannii* due to the presence of multiple class D OXA-type carbapenemases (e.g., OXA-23, OXA-51-like and OXA-48), as well as class B metallo-β-lactamase such as NDM and IMP, which are not inhibited by avibactam [55]. The presence of *bla*_NDM_ was associated with ceftazidime-avibactam-resistant in 87.3% of *K. pneumoniae* and 84% of *P. aeruginosa* isolates, regardless of the presence of *bla*_OXA−48_, *bla*_KPC_, or ESBL. Furthermore, ceftazidime-avibactam demonstrated in vitro efficacy against *K. pneumoniae*, *E. coli*, and *P. aeruginosa* isolates carrying ESBL or ESBL and other CR genes such as *bla*_OXA−48_ and *bla*_KPC_. There were however exceptions of *bla*_NDM_-positive *K. pneumoniae* and *P. aeruginosa* isolates that were susceptible to ceftazidime-avibactam, for which the phenotype loss was likely due to *bla*_NDM_ gene truncation in none-primer binding regions. These findings highlight the needs of re-evaluation of available commercial PCR systems in detecting *bla*_NDM_ gene to avoid false positives. In light of increasing molecular-based AMR gene typing data, the correlation between AMR genotype and phenotype across key antimicrobials should be continuously assessed, leveraging WGS technique. Overall, we advocate the use of commercial PCR systems for detecting carbapenem resistance genes in clinical settings to guide treatment decisions; however, the susceptibility testing results should also be considered when available.

Our study has certain limitations. Our target bacterial pathogens were likely originating from hospital-acquired infections, and thus their resistance profiles might not fully reflect the overall landscape of AMR in these pathogens. Further investigations are warranted to elucidate the role of Gram-negative bacteria in community-acquired LRTIs, particularly in lower-level healthcare settings such as provincial and district hospitals. Additionally, as we only focused on detecting commonly acquired AMR genes, resistance mechanisms driven by point mutations or efflux pump overexpression were not considered. This study primarily focused on microbiology, with limited clinical data collected and no patient follow-up after hospital discharge. Consequently, information on disease progression, treatment response, and long-term outcomes is unavailable, requiring cautious interpretation of the reported clinical outcomes.

In conclusion, we conducted a multi-hospital surveillance of LRTIs in Vietnam, highlighting the preponderance of Gram-negative bacterial pathogens, including *A. baumannii*, *K. pneumoniae* and *P. aeruginosa*, with a remarkably high MDR prevalence. Our study provides insights into the correlation between AMR genotype and phenotype for ceftazidime-avibactam resistance in *K. pneumoniae* and *P. aeruginosa* isolates, revealing a resistance level of more than 30%, largely driven by the presence of *bla*_NDM_ gene. Notably, we found *bla*_NDM_ gene truncation is not uncommon, raising concerns about the sensitivity of commercial PCR-based assays. To mitigate the impact of AMR pathogens, hospital surveillance should be further strengthened through the integration of molecular techniques to track resistant organisms, identify emerging resistance mechanisms, and support outbreak detection and response.

## Supplementary Information

Supplementary Material 1.

## Data Availability

The raw Illumina data of the study are deposited in the European Nucleotide Archive (Project Number: PRJEB86571).
