# Supplementary material for: The bacterial etiology and antimicrobial susceptibility of lower respiratory tract infections in Vietnam
Source: Ann Clin Microbiol Antimicrob. 2025 Aug 31;24:50. doi: 10.1186/s12941-025-00818-3 (PMC12400722; doi:10.1186/s12941-025-00818-3)
Supplement: Supplementary file 1 — Supplementary Material 1. [file 12941_2025_818_MOESM1_ESM.docx]

**Supplementary Tables and Figures**

Table S1. In vitro activity of ceftazidime-avibactam and comparators against carbapenem-resistant Gram-negative bacteria

Supplementary Figure 1. MIC distribution of key antibiotics against *A. baumannii* isolates

Supplementary Figure 2. MIC distribution of key antibiotics against *E. coli* isolates LRTI

Supplementary Figure 3. MIC distribution of key antibiotics against *K. pneumoniae* isolates

Supplementary Figure 4. MIC distribution of key antibiotics against *P. aeruginosa* isolates

**Table S1. In vitro activity of ceftazidime-avibactam and comparators against carbapenem-resistant Gram-negative bacteria**

|  | **Interpretation (%)** | |  | **MIC (mg/l)** | | | |  |
| --- | --- | --- | --- | --- | --- | --- | --- | --- |
|  | **N** | **Susceptible** | **N** | **MIC Range*** | **MIC50** | **MIC90** | **MIC Mode**** | **Skewness Score** |
| ***Acinetobacter baumannii*** | | | | | | | | |
| Ceftazidime/Avibactam | 150 | 1 (0.7%) | 150 | 3 to 256 | ≥256 | ≥256 | ≥256 (61/150) | -0.92 |
| Ceftazidime | 474 | 7 (1.5%) | 314 | 4 to ≥64 | ≥64 | ≥64 | ≥64 (307/314) | -7.13 |
| Imipenem | 471 | 0 (0%) | 315 | ≤8 to ≥16 | ≥16 | ≥16 | ≥16 (311/315) | -12.43 |
| Meropenem | 461 | 0 (0%) | 252 | 8 to ≥16 | ≥16 | ≥16 | ≥16 (249/252) | -11.09 |
| Colistin | 474 | 461 (97.3%) | 474 | <0.062 to 32 | 0.5 | 1 | 0.5 (207/474) | 7.6 |
| ***Escherichia coli*** | | | | | | | | |
| Ceftazidime/Avibactam | 2 | 0 (0%) | 2 | ≥256 to ≥256 | ≥256 | ≥256 | ≥256 (2/2) |  |
| Ceftazidime | 1 | 0 (0%) | 1 | ≥64 to ≥64 | ≥64 | ≥64 | ≥64 (1/1) |  |
| Imipenem | 2 | 0 (0%) | 1 | ≤16 to ≥16 | ≥16 | ≥16 | ≥16 (1/1) |  |
| Meropenem | 2 | 0 (0%) | 1 | ≤16 to ≥16 | ≥16 | ≥16 | ≥16 (1/1) |  |
| Colistin | 2 | 0 (0%) | 2 | 0.125 to 0.19 | 0.125 | 0.125 | 0.125 (1/2) |  |
| ***Klebsiella pneumoniae*** | | | | | | | | |
| Ceftazidime/Avibactam | 121 | 57 (47.1%) | 121 | 0.25 to ≥256 | 1.5 | ≥256 | ≥256 (58/121) | -0.07 |
| Ceftazidime | 108 | 3 (2.8%) | 77 | 16 to ≥64 | ≥64 | ≥64 | ≥64 (76/77) | -8.6 |
| Imipenem | 121 | 0 (0%) | 66 | 1 to ≥16 | ≥16 | ≥16 | ≥16 (57/56) | -2.36 |
| Meropenem | 113 | 0 (0%) | 66 | 4 to ≥16 | ≥16 | ≥16 | ≥16 (65/66) | -7.94 |
| Colistin | 91 | 70 (76.9%) | 91 | 0.094 to 64 | 0.125 | 12 | 0.125 (42/91) | 3.29 |
| ***Pseudomonas aeruginosa*** | | | | | | | | |
| Ceftazidime/Avibactam | 119 | 59 (49.6%) | 119 | 0.75 to ≥256 | 8 | ≥256 | ≥256 (51/119) | 0.18 |
| Ceftazidime | 118 | 16 (13.6%) | 37 | 4 to ≥64 | ≥64 | ≥64 | ≥64 (30/37) | -1.64 |
| Imipenem | 114 | 1 (0.9%) | 39 | 16 to ≥16 | ≥16 | ≥16 | ≥16 (38/39) |  |
| Meropenem | 105 | 0 (0%) | 40 | 8 to ≥16 | ≥16 | ≥16 | >=16 (39/40) | -6.08 |
| Colistin | 97 | 96 (99%) | 98 | 0.094 to 4 | 1 | 1.5 | 1 (42/97) | 2.76 |
| *** Min; Max | | | | | | | | |
| **** MIC mode (Number of cases/ Total cases) | | | | | | | | |

**
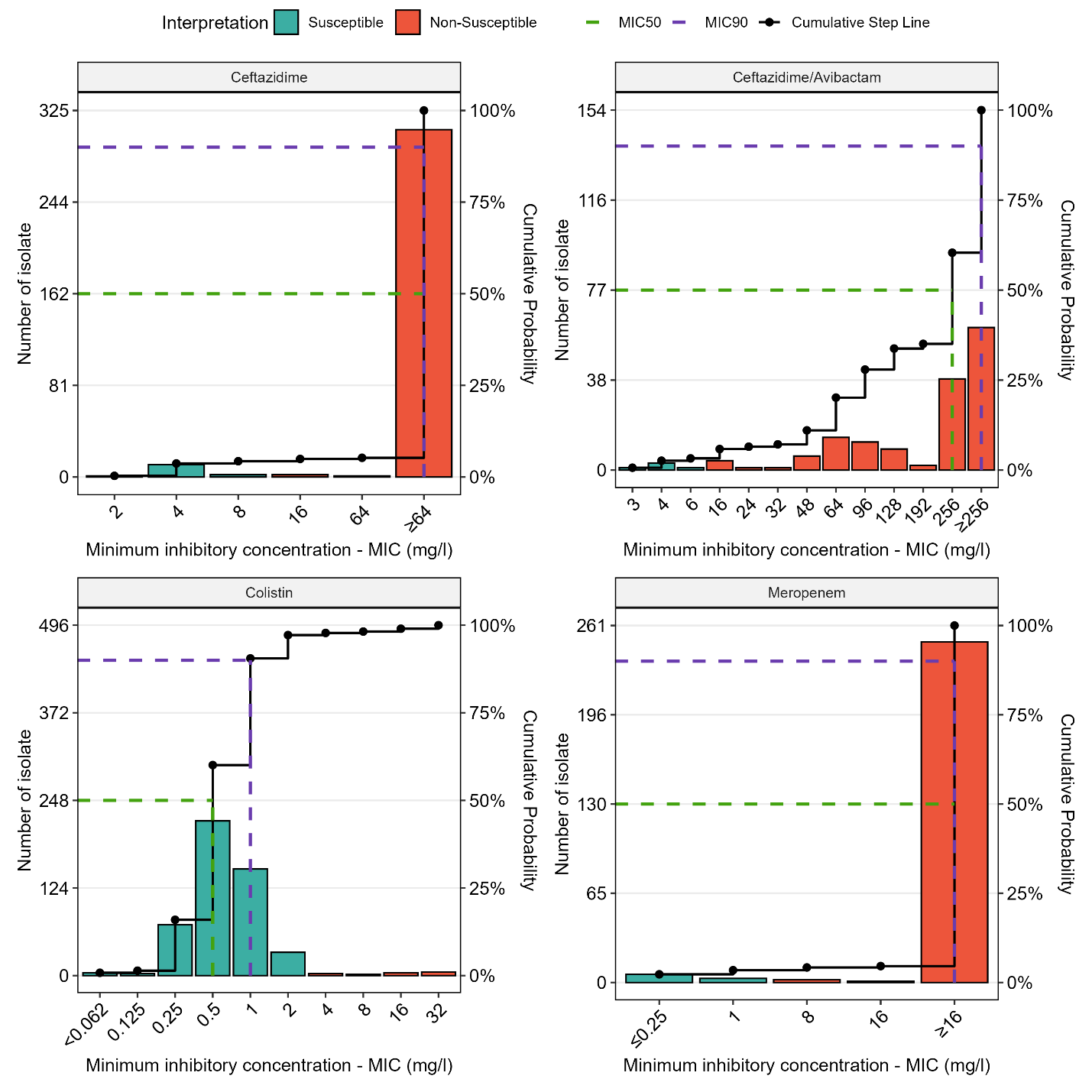
**

**Supplementary Figure 1. MIC distribution of key antibiotics against *A. baumannii* isolates**


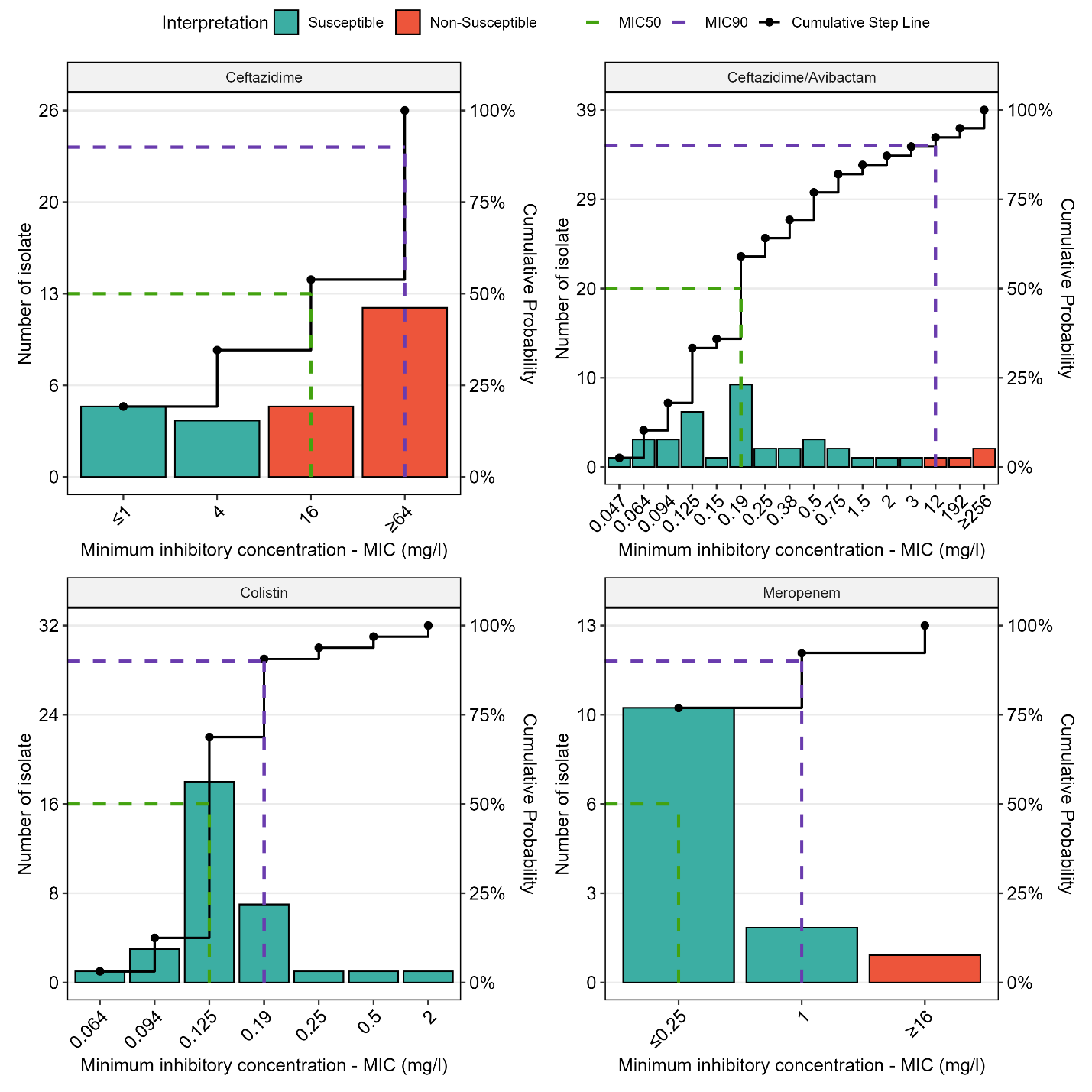


**Supplementary Figure 2. MIC distribution of key antibiotics against *E. coli* isolates**


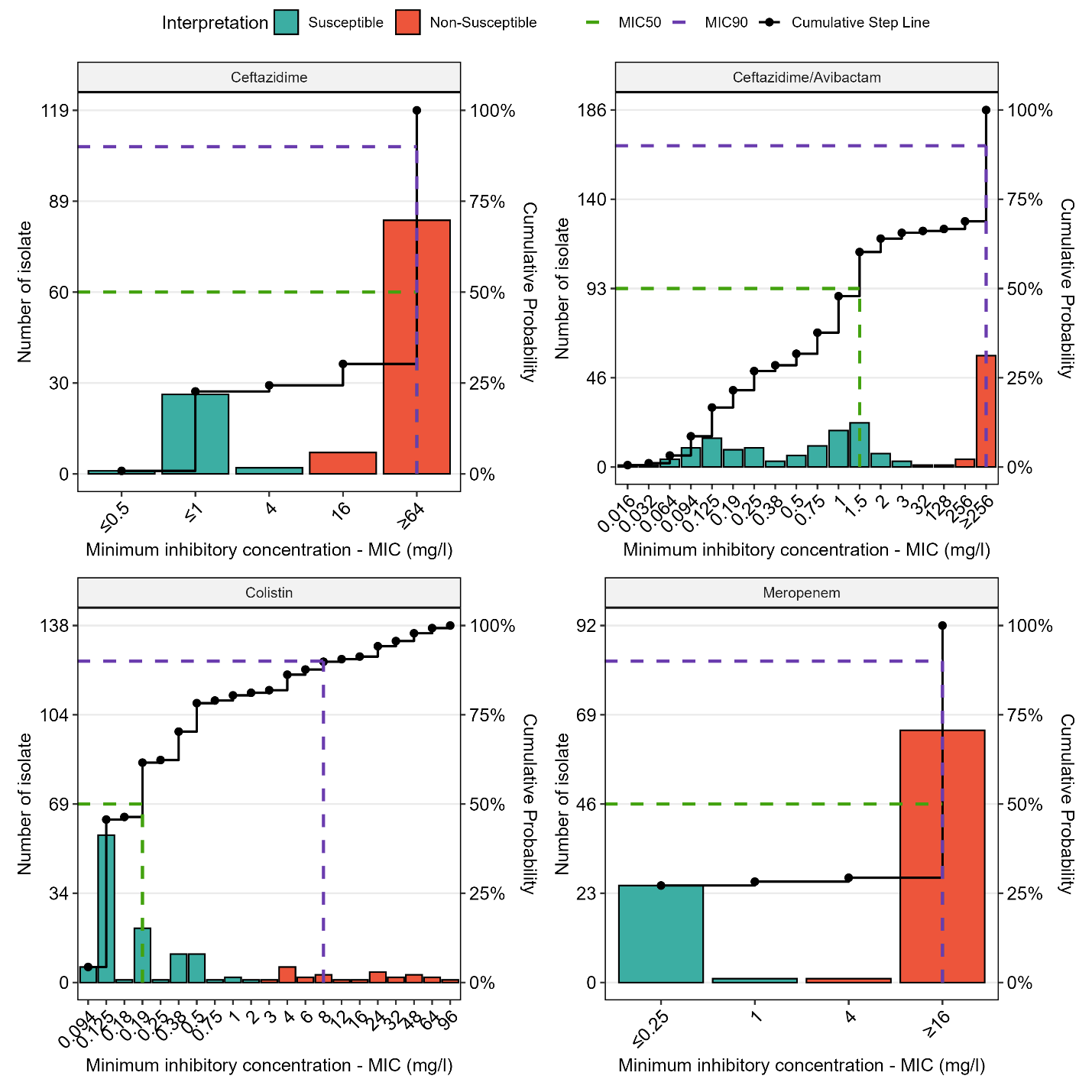


**Supplementary Figure 3. MIC distribution of key antibiotics against *K. pneumoniae* isolates**

**
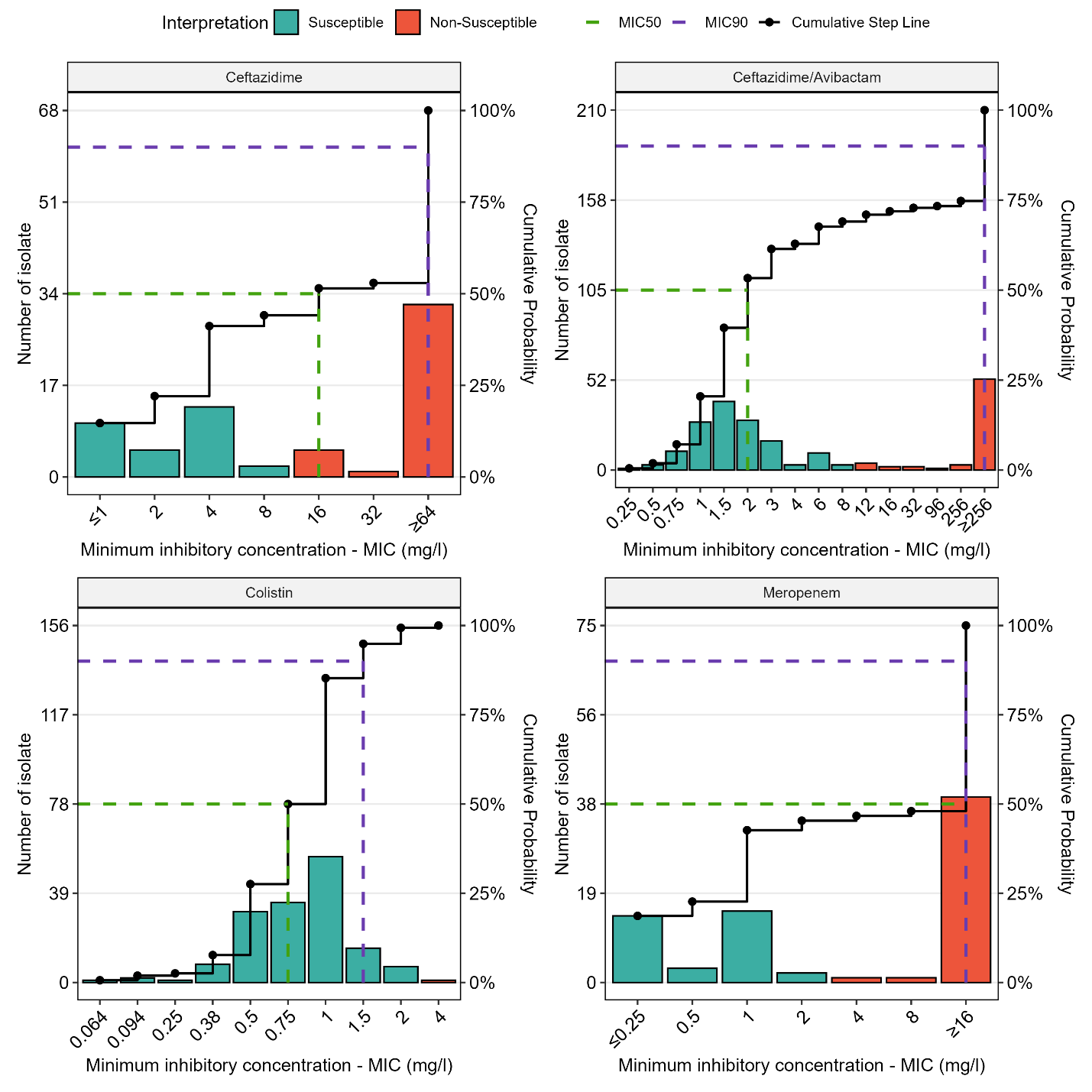
**

**Supplementary Figure 4. MIC distribution of key antibiotics against *P. aeruginosa* isolates**
